# Targeting Autophagy in Cancer: Update on Clinical Trials and Novel Inhibitors

**DOI:** 10.3390/ijms18061279

**Published:** 2017-06-16

**Authors:** Cynthia I. Chude, Ravi K. Amaravadi

**Affiliations:** Department of Medicine and Abramson Cancer Center, University of Pennsylvania, 852 BRB, 421 Curie Blvd, Philadelphia, PA 19104, USA; Cynthiachude@ymail.com

**Keywords:** autophagy, Hydroxychloroquine, lysosomes, cancer, clinical trials, retinopathy, potent autophagy inhibitors

## Abstract

Eukaryotes use autophagy as a mechanism for maintaining cellular homeostasis by degrading and recycling organelles and proteins. This process assists in the proliferation and survival of advanced cancers. There is mounting preclinical evidence that targeting autophagy can enhance the efficacy of many cancer therapies. Hydroxychloroquine (HCQ) is the only clinically-approved autophagy inhibitor, and this systematic review focuses on HCQ use in cancer clinical trials. Preclinical trials have shown that HCQ alone and in combination therapy leads to enhancement of tumor shrinkage. This has provided the base for multiple ongoing clinical trials involving HCQ alone and in combination with other treatments. However, due to its potency, there is still a need for more potent and specific autophagy inhibitors. There are multiple autophagy inhibitors in the pre-clinical stage at various stages of development. Additional studies on the mechanism of HCQ and other autophagy inhibitors are still required to answer questions surrounding how these agents will eventually be used in the clinic.

## 1. Introduction

Macroautophagy or simply autophagy helps cells maintain homeostasis and adapt to stressful conditions [1]. It is one of the three distinct autophagic pathways in mammalian cells, which includes microautophagy and chaperone-mediated autophagy [2,3]. Dysfunction of autophagy has been associated with various disorders including inflammation and neoplastic conditions, as well as neurodegenerative diseases [2,4]. Autophagy can be selective or nonselective in the removal of misfolded proteins, damaged organelles and in the elimination of intracellular viruses and pathogens [4]. Autophagy uses the autophagosome (a double membrane vesicle) to bring engulfed cytoplasmic material to the lysosome [4,5]. The autophagosome fuses with the lysosome, forming an autophagolysosome. The engulfed content inside the autophagosome is degraded by activated lysosomal hydrolases. These newly-degraded materials are recycled out of the lysosome and back to the cytosol for new biosynthesis and metabolic processes. The majority of free lysosomes in the cell is used up during the formation of autolysosomes for autophagy and is restored through autophagic lysosome reformation (ALR) [6]. During ALR, reformation tubules extrude from autolysosomes, and small vesicles made of lysosomal membrane components bud from the reformation tubules [6,7]. These small proto-lysosome vesicles are initially pH-neutral, containing autolysosomal membrane components, but lacking components from autophagosomes [6]. The proto-lysosomes eventually go through a maturation process, acquiring acidity and lysosomal luminal degradative proteins, becoming functional lysosomes [6,7].

This recycling feature of autophagy enables the progression and survival of established tumors [8]. Autophagy has a context-dependent role in cancer, by first suppressing the initiation of tumor growth at the early stages of cancer. At the later stages of established cancers, the autophagy process leads to improved survival of tumors from metabolic stresses, such as hypoxia, and therapeutic stresses, such as chemotherapy and radiotherapy [3]. Multiple studies have shown that some tumors may be autophagy-dependent, meaning that their survival is dependent on their ability to use the autophagy pathway as a source of nutrient replenishment [9]. These findings have led to the idea of using autophagic inhibitors as a new form of cancer therapy treatment [8,10].

**Figure 1:**
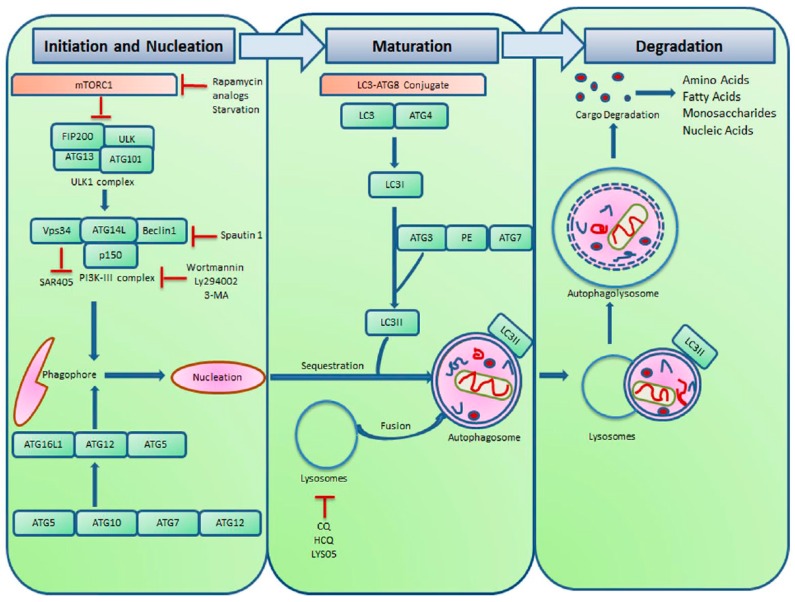
Schematic overview of the autophagic pathway and potential drug target points of the pathway. The autophagic pathway involves the following steps: induction, nucleation, maturation and degradation. Initiation of the pathway begins with growth factors signaling the activation of the mammalian target of rapamycin (mTORC1). The Unc-51-like kinase 1 (ULK1) complex consisting of autophagy-related gene 13 (ATG13) and family interacting protein of 200 Kd ( FIP200) is required to initiate Beclin 1 class III PI3K complex, which is responsible for initiating the Vacuolar Protein Sorting Protein 34 (Vps34), Beclin 1, ATG14L, p150 complex that initiates the formation of the phagophore membrane. ATG12-ATG5 and the microtubule-associated protein light chain 3 (LC3) conjugates are essential for the formation of the autophagosome and its degradation in the lysosome.

The autophagic pathway can be distinctly broken down into initiation, nucleation, autophagic vesicle maturation and the fusion and degradation of autophagic vesicle (AV) contents in the lysosomes (Figure 1) [5,11]. Many of these steps can be targeted with inhibitory drugs. In the initiation step, mTORC1 (mechanistic target of rapamycin complex 1) can be targeted because it controls the initiation of autophagic vesicle formation [5,9]. Activation of mTORC1 inhibits autophagy via the regulation of autophagy-related gene 1(ATG1) activity in response to nutrient availability [12]. Both ATG1 and ATG13 are phosphorylated under nutrient-rich conditions, which decreases the interaction of ATG13 with ATG1/uncoordinated 51-like kinase 1 (ULK1) [12]. ATG13 is rapidly dephosphorylated during starvation [12,13]. mTORC1 also inhibits autophagy by regulating the phosphorylation of the vacuolar protein sorting 34-Beclin 1 complex (Vps34) [13,14].

The nucleation step of the AV formation involves a family of phosphoinositide3-kinases (PI3Ks) [5]. Vps34 is a class III PI3K family member and regulates autophagy by generating phosphoinositol 3-phosphate, which is found in cell membranes and helps recruit a range of proteins involved in the trafficking of proteins to the membranes [14,15,16]. The interaction of Vps34 with the essential autophagy gene *beclin1* is critical for autophagosome biogenesis, maturation and apoptosis [16,17]. Beclin 1 has an anti-apoptotic role in chemotherapy, irradiation, immunotherapy, nutrient deprivation, angiogenesis inhibitors and hypoxia [17]. Inhibition of this step with PI3K inhibitors, such as 3-methyladenine (3-MA), wortmannin and LY294002, or with Vps34 inhibitors, such as SAR405, prevents the formation of autophagy vesicles [18,19,20,21,22,23]. However, at higher doses, less specific and potent agents such as 3-MA and wortmannin will inhibit class I PI3K, thereby paradoxically activating autophagy [18,24].

A third step in the maturation of AVs that could be targeted is the lipidation of microtubule-associated protein light chain 3 (LC3) [5,25]. LC3 is an ATG8 family member and is cleaved by ATG4, priming it as an ubiquitin-like substrate that can be attached to phosphatidylethanolamine (PE) in the membranes of forming autophagic vesicles. This unique lipidation of LC3 occurs via ATG12–ATG5 and E1–E3-like cascade directed by ATG3 and ATG7 [26]. ATG3 is an enzyme similar to E2 enzyme in the ubiquination pathway that catalyzes the conjugation of ATG8 and PE, a process that is necessary for the proper function of autophagy. ATG7 and ATG10 are E1- and E2-like enzymes required in the ubiquitin-like reaction between ATG5 and ATG12 [26]. ATG5-ATG12 controls the formation of autophagosomes through the LC3-II complexes. ATG8/LC3 is cleaved at the C-terminus by ATG4 protease to generate cytosolic LC3-I [26,27]. The cytosolic LC3 is conjugated to phosphatidylethanolamine to form LC3-phosphatidylethanolamine conjugate (LC3-II), which is recruited to the autophagosomal membranes where it enables autophagic vesicle growth and cargo recruitment [5,28]. Autophagosomes fuse with lysosomes to form autolysosomes, and intra-autophagosomal cargos are degraded by lysosomal hydrolases [28]. Drugs preventing the proper function of lysosomal hydrolases also lead to the accumulation of autophagic vesicles [2,5].

There are multiple compounds that inhibit the different phases of autophagy, and while drug development against these and other upstream targets continues, the only clinically-approved autophagy inhibitor is an anti-malarial chloroquine (CQ) and its derivatives, such as hydroxychloroquine (HCQ) [29]. HCQ can inhibit lysosomal acidification and prevent the degradation of autophagosomes, thereby suppressing autophagy [30,31]. The mechanism by which CQ derivatives interfere with autophagy is still not very well understood [30]. It could be acting simply as a weak base that gets transported and trapped inside the lysosome, de-acidifying the lysosome, or it could be interfering with a specific protein function or production [30]. CQ derivatives have also been shown to function by releasing anti-cancer lysotrophic drugs from the lysosome. Lysotrophic drugs are easily trapped into the lysosomes, but when combined with CQ derivatives, the lysosomal trapping of these drugs is reduced, increasing the concentration of the drugs in the cytoplasm [32,33].

For clinical trials, HCQ was chosen over CQ as an autophagy inhibitor because it is less toxic than CQ at peak concentrations [34,35,36,37]. HCQ has been shown to have antineoplastic effects in numerous preclinical experiments when combined with other agents [38]. HCQ inhibits autophagy by preventing the lysosome from degrading and recycling the materials engulfed in the autophagosome [37,39]. This review will discuss HCQ preclinical and clinical trials, with special attention paid to dosage and side effects. We will also discuss the preclinical studies of other autophagy inhibitors such as verteporfin and lys05, which have clinical potential [39,40].

## 2. Hydroxychloroquine Clinical Trials

Preclinical studies with HCQ in tumor cell lines and animal models have provided the premise of inhibiting autophagy to overcome chemotherapeutic resistance. In renal cell carcinoma lines, HCQ enhanced the cytotoxicity of temsirolimus, promoting apoptosis and causing the downregulation of phospho-S6 through a mechanism not found in other autophagy inhibitors, such as bafilomycin A1 [41]. In breast cancer cells, the combination of HCQ and tamoxifen (TAM) was more effective at inhibiting autophagy than monotherapy in estrogen receptor-positive (ER^+^) breast cancer cell lines [29]. The successful results of these and recent preclinical investigations of in vivo and in vitro studies with HCQ provided the rationale for launching multiple clinical trials. The first of these clinical trials was done by Wolpin et al., but it recruited patients with an Eastern Cooperative Oncology Group performance status (ECOG ps) of 2, instead of the typical patients enrolled into clinical trials with ECOG ps of 0 or 1 [42]. They studied the safety and antineoplastic activity of HCQ in 20 patients with metastatic pancreatic cancer that did not respond to conventional chemotherapy [42]. In this phase II clinical trial, patients received 400 (*n* = 10) or 600 (*n* = 10) mg HCQ twice daily as a single therapeutic agent [42]. Though these dosages where well tolerated, the study had two patients whom developed treatment-related Grade 3/4 side effects [42]. The results of six other phase I clinical trials using HCQ as an autophagy inhibitor were published in 2014 [34,43,44,45,46,47]. These trials involved HCQ in combination with various cancer chemotherapy and targeted therapies. Five of the trials were done in humans and one in pet dogs with spontaneous lymphoma, to determine the safety and engagement of HCQ treatment with the autophagy pathway. The therapeutic regimens involved HCQ in combination with either temozolomide, bortezomib, temsirolimus, vorinostat or doxorubicin. A number of patients with melanoma, colorectal cancer, myeloma and renal cell carcinoma showed partial response and stable disease, suggesting that in a subset of patients, HCQ had significant antitumor activity in patients [34,43,44,45,47].

These studies were the first to show that high doses of HCQ could be combined safely with other cytotoxic chemotherapies. Rangwala et al. showed that when HCQ was combined with dose-intense temozolomide (oral alkylating agent), 1200 mg HCQ daily (600 mg twice a day), which was the highest dosage allowed by the food and drug administration, were well tolerated without the production of excess toxicity [44]. Preclinical studies preceding this trial showed that autophagy may play a role in limiting the efficacy of alkylating chemotherapy [44]. In this phase I trial, no recurrent dose-limiting toxicities were observed when 200–1200 mg HCQ were combined with 150 mg/m^2^ of temozolomide, in patients with advanced solid malignancies [44]. Overall, a 14% partial response and 27% stable disease rate in patients with wild-type (serine/threonine-protein kinase B-raf) BRAF melanoma was observed [44]. A phase I dose-escalation study with 27 patients to evaluate the maximum tolerated dose and safety of combining temsirolimus (rapamycin analog) with HCQ was also performed by Rangwala et al. [45]. In preclinical studies, combining HCQ with a rapamycin analog was found to produce synergistic antitumor activity in animal models, by simultaneously suppressing mTORC1 activity, thereby inducing autophagy and blocking effective autophagic flux with HCQ at the level of the lysosome [45]. Temsirolimus in combination with HCQ was well tolerated with 7% anorexia, 7% fatigue and 7% nausea. Sixty-seven percent of patients in the dose-escalation study achieved stable disease, with evidence of autophagy inhibition in serial peripheral blood mononuclear cells and tumor biopsies in patients treated with 1200 mg of HCQ daily [45]. Vogl et al. were able to increase the efficacy of proteasome inhibition for myeloma by combining bortezomib (proteasome inhibitor) and HCQ in a phase I trial [43]. Myeloma plasma cells appear to be susceptible to proteasome inhibition, and preclinical studies suggest that the efficacy of proteasome inhibitor therapy depends on the ability of malignant plasma cells to degrade misfolded proteins [43]. Combined proteasome and autophagy inhibition therefore results in the accumulation of misfolded proteins and synergistic cytotoxicity [43]. Twenty five patients were enrolled in the combination therapy of HCQ and bortezomib, and the most common adverse event was Grade 2 gastrointestinal toxicity and cytopenias. Forty five percent of patients showed stable disease as their best response, although some of these patients had not had prior bortezomib [43].

Though the previously mentioned studies did not reach a maximum tolerated dosage (MTD) of HCQ, other studied, such as Rosenfeld et al., were able to observe that at 800 mg of HCQ daily, many subjects experienced Grade 3 and 4 neutropenia and thrombocytopenia in combination therapy with temozolomide (alkylating agent) [46]. In this study, HCQ (at the dose below the MTD: 600 mg/d when combined with low dose continuous temozolomide showed no significant improvement in overall survival in patients with malignant glioma [46]. Mahalingam et al. also showed dose-associated fatigue and gastrointestinal dose-limiting toxicities, but at 600 mg of HCQ daily, when combined with histone deacetylases (HDACs) inhibitor vorinostat [47]. HDACs regulate the removal of acetyl groups, leading to the repression of transcription activity [48]. Cancer cells overexpress HDAC proteins to promote malignant progression and anticancer drug resistance [47,48]. Out of 27 patients treated, Mahalingam et al. considered 24 to be fully evaluable for study assessments. They observed durable partial response in one patient with renal cell carcinoma and prolonged stable disease in two patients with colorectal cancer [47]. In both Rosenfeld et al. and Mahalingam et al., the dose-limiting toxicities were known toxicities of temozolomide and vorinostat, respectively. In contrast to HCQ combination studies, Wolpin et al., in a phase II trial on patients with pancreatic cancer, observed negative efficacy with HCQ treatment as a single agent [42]. Though they were able to observe tolerance at high HCQ dosages, their results suggest that autophagy inhibitors in combination with HCQ therapy are better at producing positive anti-cancer therapy results than HCQ therapy alone [42].

The use of hydroxychloroquine at low doses and in short time periods shows rare occurrences of side effect [5]. However, at higher doses and longer duration, some serious side effects have been observed [40]. One of these side effects is retinal toxicity [44]. Retinal toxicity from high dose and duration of HCQ usage has been on the rise [8]. Multiple studies have shown that retinopathy was unlikely to occur with HCQ at dosages less than 6.5 mg/kg body weight within the first 5–10 years of therapy [45]. The risk of the toxicity is less than 1% in the first five years and less than 2% up to 10 years [8]. Annual screening after five years of HCQ usage is recommended for patients to detect early changes in the retina before vision is compromised [8].

Though these clinical studies have helped answer multiple questions concerning autophagy cancer therapy, there are still many questions left to be answered. One of these questions involves finding a better reliable biomarker for autophagy detection. In the combination study with HCQ and vorinostat, Mahalingam et al. show that analysis of peripheral blood mononuclear cells may not be an ideal biomarker of autophagy inhibition [47]. Currently, microtubule-associated protein light chain 3 (LC3) is the only protein widely used as a marker for autophagosomes due to its existence on autophagosomes [49]. However, it is important to note that monitoring the increase and decrease of autophagosomes in cells does not directly correspond to the increase or decrease in cellular autophagic activity [49]. The number of autophagosomes observed at any given time point is either due to autophagy induction or suppression of various steps in the autophagy pathway [49]. One of the important questions also left to be determined is what impact autophagy inhibition will have on the toxicity of radiotherapy and whether autophagy induced by radiation therapy should actually be suppressed, because it is not completely know if it could be causing more harm than benefit to the patients. Additional questions for HCQ studies include: Which tumors should be targeted for this approach? Can a biomarker strategy help? Hopefully, these questions can be answered in the current clinical trials in Table 1 and in future clinical trials.

## 3. New Potent Autophagy Inhibitors

HCQ is the only clinically-approved autophagy inhibitor, but the pharmacodynamic studies indicate that higher doses of HCQ up to 1200 mg/d produce only modest inhibition in vivo and can be inconsistent [10]. It also fails to inhibit autophagy in acidic environments around 6.5, due to the reduction in the cellular uptake of the drug in these environments [10,50]. The limitation of HCQ’s ability to function in vivo creates a demand for the production of more potent autophagy inhibitors. Table 2 summarizes these inhibitors and their target point in the autophagic pathway.

SBI-0206965 is a small molecule inhibitor of the autophagy kinase ULK1 [51]. SBI-0206965 suppressed ULK-1-mediated phosphorylation events in cells regulating autophagy [51]. Egan et al. show that when synergized with the mTOR inhibitor rapamycin, SBI-0206965 produced autophagy inhibition, leading to the death of tumor cells [51]. SBI-0206965 should be considered a potential autophagy inhibitor when targeting ULK1/ATG1.

Spautin-1 is a novel autophagy inhibitor that causes an increase in proteasomal degradation of class III PI3 kinase complexes [15]. It promotes the degradation of PI3 kinase complexes by inhibiting USP10 and USP13, which are ubiquitin-specific peptidases that target the Beclin 1 subunit of Vps34 complexes [52]. Shao et al. show that spautin-1 inhibits imatinib mesylate-induced autophagy in chronic myeloid leukemia cells by downregulating Beclin 1 [53]. They also show that the pro-apoptotic activity of spautin-1 was associated with GSK3β, which is an important downstream effector of PI3k/Akt [53]. These results indicate a potential therapeutic application for the spautin-1 inhibitory effect in cancer cells.

SAR405 is a kinase inhibitor of Vps18 and Vps34 [22]. Inhibiting Vps34 resulted in lysosomal function impairment, affecting vesicle trafficking between late endosome and the lysosome [54]. The combination of SAR405 with everolimus worked together to enhance anti-proliferation activity in renal cancer cell lines [22]. This shows a potential cancer therapy application for Vps34 inhibitors in cancer.

ATG4 inhibitors such as NSC185058 and NSC377071 have been used to inhibit ATG4B, LC3 lipidation and autophagy [55]. NSC185058 has been shown to suppress tumor growth in osteosarcoma without affecting the mTOR or PtdIns3K (class III phosphatidylinositol 3-kinase) pathways [52]. ATG4B is a cysteine protease that activates LC3 lipidation and recycles lipidated LC3 to reform free LC3 [55]. Akin et al. show that NSC185058 inhibits the C-terminal cleavage of LC3B by ATG4B in rapamycin-treated 293T cells and in amino acid-starved differentiated hepatocyte derived cellular carcinoma line (HuH7 cells), demonstrating that NSC185058 effectively inhibits ATGB activity in vitro and in vivo [55]. ATGB antagonists should be considered as useful autophagy inhibitors for treatment of aggressive cancers, such as osteosarcoma.

Verteporfin, an approved agent for photodynamic therapy, inhibits autophagosome formation. Verteporfin inhibits autophagy at an early stage and does not cause autophagosome accumulation, unlike HCQ, which functions on the lysosome and leads to autophagosomal accumulation [56]. Donohue et al. show that verteporfin moderately enhances the antitumor activity of gemcitabine in a pancreatic ductal adenocarcinoma model [39]. Gemcitabine is an autophagy inducer used as an antineoplastic chemotherapy drug [57]. Donohue et al. show that verteporfin enhances gemcitabine anti-tumor activity by inhibiting autophagy through the inhibition of gemcitabine-induces p62 degradation and increasing the accumulation of LC3-II [39]. Verteporfin does not reduce tumor volume as a single agent, but should be considered a potential pancreatic ductal adenocarcinoma autophagy inhibitor when combined with gemcitabine.

ROC325 is a dimeric compound containing modified HCQ and lucanthone scaffolds [58]. It is water soluble and exhibits superior lysosome autophagic inhibition compared to HCQ [58]. In vitro and in vivo studies in renal cell carcinoma showed that ROC325 induced the accumulation of autophagosomes with un-degraded cargo, lysosomal deacidification, P62 stabilization and disruption of autophagic flux, significantly better than HCQ [59].

Lys05 is a water-soluble analog of HCQ [8]. It has been identified as a new lysosomotropic agent that at a low daily dose elevates the pH of the lysosome and blocks autophagy [45]. Unlike HCQ, lys05 produces more potent antitumor activity as a single agent in vitro and in vivo in two melanoma xenograft models and a colon cancer xenograft model compared to HCQ [34]. The increased potency of lys05 can be associated with its bivalent aminoquinoline rings and carbon 7 chlorine [45]. The combination of lys05 with a BRAF inhibitor had significant inhibition activity in vivo [46]. Lys05 was also shown to enhance the antitumor activity of receptor tyrosine kinase inhibitors, such as sunitinib, in clear cell ovarian cancer [47]. Lys05 is a new lysosomal autophagy inhibitor that is more potent than HCQ, with the potential to be developed further into a clinical autophagy inhibitor. The development of these more specific autophagy inhibitors provides for better anti-cancer treatment opportunities.

## 4. Conclusions

HCQ is an effective and safe cancer therapy drug, but additional mechanistic studies in preclinical models are still required to better understand its target molecule [61]. It has some dose-sensitive effects, and a number of molecular modifications, such as HCQ nanocarriers, have been used to improve its pharmacokinetic and pharmacodynamics properties, in order to reduce the undesirable side effect of the drug [10]. HCQ’s inhibitory interaction with the lysosome could be contributing to defective lysosomes function, which has been known to cause lysosomal storage disease [62]. More specific autophagy inhibitors are being developed that may prove to be better at augmenting chemotherapy treatments compared to HCQ. Some future directions for the improvement of autophagy-associated cancer therapy include better understanding of the two-faced role of autophagy in tumor survival, the development of a molecular marker for autophagic flux in human tumors, the identification of patient subpopulations that will be most susceptible to autophagy inhibition and better understanding of the interaction of the immune system with tumor cells.

## Figures and Tables

**Table 1:** Current Hydroxychloroquine (HCQ) clinical trials.

Treatment	Condition	Phase	Trial Reference # at ClinicalTrials.gov
HCQ + sunitinib malate	Adult solid neoplasm	I	NCT00813423
HCQ + vorinostat	Malignant solid tumor	I	NCT01023737
HCQ + sirolimus or vorinostat	Advanced cancers	I	NCT01266057
HCQ + Protein kinase B (Akt) inhibitor MK-2206 dihydrochloride (MK2206)	Advanced cancers	I	NCT01480154
HCQ as a single agent	Estrogen receptor positive breast cancer	I	NCT02414776
HCQ + gemcitabine	Advanced adenocarcinoma	I/II	NCT01506973
HCQ + Interleukin 2(IL-2)	Renal cell carcinoma	I/II	NCT01550367
HCQ + vorinostat	Colorectal cancer	I/II	NCT02316340
HCQ + gemcitabine/carboplatin	Small cell lung cancer	I/II	NCT02722369
HCQ + capecitabine	Pancreatic carcinoma	II	NCT01494155
HCQ as a single agent	Prostate cancer	II	NCT00726596
HCQ + Abraxane and gemcitabine	Pancreatic carcinoma	II	NCT01978184

**Table 2:** Autophagy inhibitors.

Name	Mechanism	Target Point	References
3-Methyladenine	phosphoinositide3-kinase (PI3) inhibitor	Autophagosome formation	[18]
Wortmannin	PI3-kinase inhibitor	Autophagosome formation	[19]
LY294002	PI3-kinase inhibitor	Autophagosome formation	[20]
SBI-0206965	Unc-51-like kinase 1 (ULK1) Inhibitor	Autophagosome formation	[51]
Spautin-1	ubiquitin-specific peptidases (USP10) and (USP13) inhibitor	Autophagosome formation	[15,52,53]
SAR405	Vacuolar Protein Sorting Protein 18 and 34 (Vps18 and Vps34) inhibitor	Autophagosome formation	[22,54]
NSC185058	autophagy-related gene 4 (ATG4) inhibitor	Autophagosome formation	[55]
Verteporfin	Unknown	Autophagosome formation and accumulation	[39,56]
ROC325	Unknown	Lysosome	[58,59]
Lys05	Unknown	Lysosome	[8,36,47,48,49]
Chloroquine	Unknown	Lysosome	[29,30,37]
Hydroxychloroquine	Unknown	Lysosome	[30,31,35,37,60]
